# Psychiatric Comorbidities and Outcomes in Epilepsy Patients: An Insight from a Nationwide Inpatient Analysis in the United States

**DOI:** 10.7759/cureus.1686

**Published:** 2017-09-14

**Authors:** Rikinkumar S Patel, Ahmed Elmaadawi, Zeeshan Mansuri, Mandeep Kaur, Kaushal Shah, Suhayl Nasr

**Affiliations:** 1 Department of Psychiatry, Northwell Zucker Hillside Hospital; 2 Psychiatry, Beacon Health System; 3 Psychiatry, Texas Tech University Health Science Center; 4 American University of Antigua; 5 Public Health, Western Kentucky University

**Keywords:** epilepsy, seizure, depression, psychosis, alcohol abuse, drug abuse, comorbidities

## Abstract

Background

Psychiatric comorbidities in epilepsy impose significant burdens on patients and their families. It affects their quality of life and medical care and results in cost increases. This study reports the impact of various psychiatric comorbidities in epilepsy patients regarding hospital outcomes and in-hospital mortality.

Methods

We used the Nationwide Inpatient Sample (NIS) from the Healthcare Cost and Utilization Project (HCUP) from years 2013-2014. We identified epilepsy as the primary diagnosis and psychiatric comorbidities, namely, alcohol abuse, depression, drug abuse, and psychosis, using validated International Classification of Diseases, 9th Revision, Clinical Modification (ICD-­9-­CM) codes. The differences in comorbidities were quantified using chi-square (χ^2^) tests and the multinomial logistic regression model was used to quantify associations among comorbidities using the adjusted Odds Ratio (aOR).

Results

We analyzed 397,440 hospitalizations with epilepsy as the primary diagnosis. The most prevalent psychiatric comorbidities present in epilepsy were depression (13%) followed by psychosis (10.4%). The risk of inpatient death was only seen in epilepsy with comorbid alcohol abuse (aOR 1.164; 95%CI 1.043 – 1.300; p-value =0.007). Epilepsy with comorbid depression (aOR 1.473; 95% CI 1.391 – 1.559; p-value <0.001) was associated with a higher risk of a length of stay of more than three days (median), followed by comorbid psychosis (aOR 1.290; 95% CI 1.258 – 1.322; p-value <0.001). Epilepsy with comorbid depression (aOR 1.242; 95% CI 1.172 – 1.317; p-value <0.001) was associated with a higher risk of inpatient total charge of more than $21,000 (median), followed by comorbid psychosis (aOR 1.071; 95% CI 1.045 – 1.098; p-value <0.001).

Conclusion

Psychiatric comorbidities are influential factors that must be considered in models of Health-Related Quality of Life (HRQOL) in epilepsy. Further, efforts to improve HRQOL and reduce the burden of epilepsy require greater emphasis on the early diagnosis and treatment of comorbid psychopathology.

## Introduction

Epilepsy is a diverse group of neurological disorders of variable types and severities that are characterized by recurrent seizures. A person with two or more seizures that are not provoked by specific events, such as trauma, infection, fever, or chemical change, is considered to have epilepsy [1]. Epilepsy is one of the most prevalent chronic neurologic diseases in the United States, affecting an estimated 5.1 million people (1.8% of the total population) [2-3], and the total indirect and direct cost of epilepsy is estimated to be $15.5 billion yearly [4].

Psychiatric disorders are frequently encountered in patients with epilepsy, and these may negatively influence the course of epilepsy. It can lead to an inadequate response to treatment and contribute to a reduced quality of life as well as increased mortality [5-6]. However, psychopathology is frequently unrecognized and untreated in patients with epilepsy [7]. When comorbidities are better understood, it leads to better treatment, training of physicians, and recognition of cause. This can ultimately result in improved care for epilepsy patients.

Comorbidities in epilepsy impose significant burdens on patients and their families. It affects their quality of life and medical care and results in cost increases. Comorbid conditions raise the cost of medical care through increased pharmaceutical medication use, outpatient office visits, and inpatient hospitalization [8]. To the best of our knowledge, an estimation of patient hospitalization outcomes and the costs of patient care with comorbid psychiatric disorders has not been done.

## Materials and methods

Our data came from the 2013 to 2014 Agency for Healthcare Research and Quality (AHRQ) Health Care Cost and Utilization Project (HCUP) Nationwide Inpatient Sample (NIS) [9]. In the United States, the NIS has the largest publicly available all-payer inpatient care database. The 2013-2014 NIS data contains data from a sampling of 4,411 hospitals from 45 states. This represents a pool of approximately 20% of US hospitals. We reviewed the hospital discharge data of epilepsy diagnosis by categorizing AHRQ clinical classification software diagnosis category 83 (International Classification of Diseases, 9th Edition, Clinical Modifications, ICD-9-CM, codes 345) [10]. The NIS data shows that the epilepsy condition was primarily responsible for the initial hospitalization of patients.

Assessment of comorbidities

Comorbidities are considered coexisting conditions to epilepsy and are the disorder under study. AHRQ comorbidity software was utilized to generate binary variables. Using International Classification of Diseases, 9th Edition, Clinical Modifications (ICD-9-CM) codes, this variable identified four comorbidities in the records of discharge [10]. As shown in Table 1, the ICD-9-CM codes were used for psychiatric comorbidities [10].

**Table 1:** ICD-9-CM diagnosis codes for psychiatric comorbidities ICD-9-CM: International Classification of Diseases, 9^th^ Revision, Clinical Modification

Comorbidity	ICD-9-CM Diagnosis Codes
Alcohol abuse	291.0-291.3, 291.5, 291.8, 291.81, 281.82, 291.89, 291.9, 303.00-303.93, 305.00-305.03
Drug abuse	292.0, 292.82-292.89, 292.9, 304.00-304.93, 305.20-305.93, 648.30-648.34
Psychosis	295.00-298.9, 299.10, 299.11
Depression	300.4, 301.12, 309.00, 309.1, 311

Inpatient outcomes

NIS explains death as in-hospital mortality. We calculated the length of stay as the number of nights the patient stayed in the hospital for a medical condition. We took the total discharge data that were reported in the 2013-2014 NIS and converted it to costs using hospital-specific cost to charge ratios.

Statistical analysis

The population estimated in this study is derived from the NIS database. We adjusted the means, proportions, and standard errors of the discharge weights. We used Pearson’s chi-square (χ^2^) test and the independent sample T-test for verifying and validating the continuous data, respectively. Furthermore, the categorical variables are in percentages. We also used discharge weight, which is given in the NIS database, to obtain notional inpatient data. A p-value <0.05 was used to determine the statistical significance of the test result. Differences in comorbidities were quantified using chi-square tests. We used a multinomial logistic regression model to quantify associations among comorbidities and inpatient death (adjusted Odds Ratio (aOR)), length of stay (aOR), and inpatient costs (aOR). We applied discharge weights in all regression models, and age, gender, race, median income of the patient’s zip code, hospital bed size, agency, location, region (northeast, midwest, south, and west), and teaching status were approximated. Statistical analysis was done using SPSS 22 (IBM, New York, United States) [11]. Our database did not contain patients' personally identifiable information. Therefore, Institution Review Board (IRB) was not required for this study.

## Results

Characteristics of the population

The sample consisted of 397,440 hospitalizations with epilepsy listed as the primary diagnosis, as shown in Table 2. Approximately 50.7% (n = 201,840) of epilepsy patients were male, and the average age of patients was almost 41.8 years (S.D. = 25.111). A total of 220,350 epilepsy patients were white, which comprises 58.8% of the total, followed by black (22.2%), Hispanic (12.7%), Asian or Pacific Islander (1.7%), Native American (0.6%), and others (four percent). The maximum number of hospitalizations due to epilepsy (37.7%) occurred in the southern region of the United States. The majority of the admissions for epilepsy were covered by Medicare (34.3%) and Medicaid (29.7%).

**Table 2:** Characteristics of the population Significant p-value ≤ 0.05 at 95% confidence interval, variables are Agency for Healthcare Research and Quality (AHRQ) co-morbidity measures. HMO: health maintenance organization

Variable	Estimate (proportion)	p-value
Age in years at admission		
Mean age ± SD	41.8 ± 25.111	<0.001
1-17	22.0%	<0.001
18-44	28.7%	<0.001
45-64	28.6%	<0.001
65-84	17.0%	<0.001
>85	3.7%	<0.001
Indicator of sex		
Male	50.7%	<0.001
Female	49.3%	<0.001
Race		
White	58.8%	<0.001
Black	22.2%	<0.001
Hispanic	12.7%	<0.001
Asian or Pacific Islander	1.7%	<0.001
Native American	0.6%	<0.001
Other	4.0%	<0.001
Region of hospital		
Northeast	23.5%	<0.001
Midwest	22.0%	<0.001
South	37.7%	<0.001
West	16.8%	<0.001
Primary expected payer		
Medicare	34.3%	<0.001
Medicaid	29.7%	<0.001
Private including HMO	26.4%	<0.001
Self-pay	5.5%	<0.001
No charge	0.7%	<0.001
Other	3.4%	<0.001

Inpatient outcomes in epilepsy

A total of 319,975 patients (80.7% of the total) with a primary diagnosis of epilepsy were hospitalized based on nonelective or emergency admission. Inpatient outcomes in epilepsy are shown in Table 3. The mean length of stay in the hospital was 3.83 days (p-value <0.001), and the mean inpatient total charge was $35,973.70 (p-value <0.001). During the period 2013-2014, there were 1.5 million inpatient days and $14.3 billion in inpatients costs due to epilepsy in the United States. About 72.7% (n= 289,060) patients with epilepsy were discharged back home, 14 % (n= 55,715) were discharged to skilled nursing or an intermediate care facility and 2.1% (n= 8,265) were discharged to an acute care hospital to receive further specialized care. The in-hospital mortality rate of epilepsy was low (0.7%), as only 2,755 inpatient deaths occurred during 2013-2014.

**Table 3:** Inpatient outcomes in epilepsy Significant p-value ≤ 0.05 at 95% confidence interval, variables are Agency for Healthcare Research and Quality (AHRQ) co-morbidity measures. SNF: skilled nursing facility, INF: intermediate nursing facility

Variable	Estimate	p-value
Died during hospitalization		
Did not die	99.3%	<0.001
Died	0.7%	<0.001
Length of stay		
Mean length of stay	3.83 days	<0.001
Median length of stay	3 days	<0.001
Total charges		
Mean total charges	$35973.70	<0.001
Median total charges	$21000	<0.001
Disposition of patient		
Routine	72.7%	<0.001
Short-term hospitals	2.1%	<0.001
Other (SNF, ICF, another facility)	14.1%	<0.001
Home health care	7.9%	<0.001
Against medical advice	2.5%	<0.001
Died	0.7%	<0.001
Elective vs. non-elective admissions		
Non-elective/Emergency	80.7%	<0.001
Elective	19.3%	<0.001

Psychiatric comorbidities in epilepsy

The present study shows that the pervasiveness of psychiatric comorbidity in patients with epilepsy is estimated to be 39.9%. The most prevalent psychiatric comorbidities present in epilepsy were depression (n= 51,885 (13%)) followed by psychosis (n= 41,270 (10.4%)). Alcohol abuse (n= 34,540 (8.7%)) and drug abuse (n= 30,975 (7.8%)) were less prevalent psychiatric comorbidities in epilepsy. These four psychiatric comorbidities, namely, depression, psychosis, alcohol abuse, and drug abuse, constitute a total 39.9 % proportion of the comorbidities associated with epilepsy. The prevalence of psychiatric comorbidities is shown in Table 4.

**Table 4:** Psychiatric comorbidities in epilepsy Significant p-values ≤ 0.05 at 95% confidence interval, variables were Agency for Healthcare Research and Quality (AHRQ) comorbidity measures.

Psychiatric Comorbidity	Estimate (proportion)	p-value
Alcohol abuse	8.7%	<0.001
Depression	13.0%	<0.001
Drug abuse	7.8%	<0.001
Psychosis	10.4%	<0.001

Comorbidities and inpatient outcomes

Associations among observed comorbidities and inpatient outcomes are presented in Tables 5, 6, 7. Relative to the other psychiatric comorbidities, the risk of inpatient death was only seen in epilepsy with comorbid alcohol abuse (aOR 1.164; 95% CI 1.043 – 1.300; p-value = 0.007). The mean length of stay of epilepsy patients during hospitalization was 3.83 days (median = 3 days; p-value <0.001). Relative to the other psychiatric comorbidities, epilepsy with comorbid depression (aOR 1.473; 95% CI 1.391 – 1.559; p-value <0.001) was associated with a higher risk of length of stay of more than three days (median), followed by comorbid psychosis (aOR 1.290; 95% CI 1.258 – 1.322; p-value <0.001). On the contrary, comorbid drug abuse did not have an increased risk of the length of stay of more than three days (aOR 0.833; 95% CI 0.809 – 0.856; p-value <0.001). The mean inpatient total charge for epilepsy patients during hospitalization was $35,973.70 (median = $21,161; p-value <0.001). Relative to the other psychiatric comorbidities, epilepsy with comorbid depression (aOR 1.242; 95% CI 1.172 – 1.317; p-value <0.001) was associated with a higher risk of inpatient total charge of more than $21,000 (median), followed by comorbid psychosis (aOR 1.071; 95% CI 1.045 – 1.098; p-value <0.001). On the other hand, comorbid alcohol abuse did not have an increased risk of inpatient total charge of more than $21,000 (aOR 0.926; 95% CI 0.903 – 0.0950; p-value <0.001).

**Table 5:** Psychiatric comorbidities and inpatient death Significant p-values ≤ 0.05 at 95% confidence interval, variables were Agency for Healthcare Research and Quality (AHRQ) comorbidity measures. aOR: adjusted Odds Ratio CI: confidence interval

Comorbidities	Inpatient Death	
	aOR (95% CI)	P-value
Comorbid alcohol abuse	1.164 (1.043 – 1.300)	0.007
Comorbid depression	0.540 (0.472 – 0.617)	<0.001
Comorbid drug abuse	0.926 (0.763 – 1.123)	0.435
Comorbid psychosis	0.487 (0.402 – 0.591)	<0.001

**Table 6:** Psychiatric comorbidities and length of stay Significant p-values ≤ 0.05 at 95% confidence interval, variables were Agency for Healthcare Research and Quality (AHRQ) comorbidity measures. aOR: adjusted Odds Ratio CI: confidence interval

Comorbidities	Length of Stay	
	aOR (95% CI)	p-value
Comorbid alcohol abuse	1.037 (1.010 – 1.064)	0.007
Comorbid depression	1.473 (1.391 – 1.559)	<0.001
Comorbid drug abuse	0.833 (0.809 – 0.856)	<0.001
Comorbid psychosis	1.290 (1.258 – 1.322)	<0.001

**Table 7:** Psychiatric comorbidities and hospitalization cost Significant p-values ≤ 0.05 at 95% confidence interval, variables were Agency for Healthcare Research and Quality (AHRQ) comorbidity measures. aOR: adjusted Odds Ratio CI: confidence interval

Comorbidities	Hospitalization Cost	
	aOR (95% CI)	P-value
Comorbid alcohol abuse	0.926 (0.903 – 0.950)	<0.001
Comorbid depression	1.242 (1.172 – 1.317)	<0.001
Comorbid drug abuse	0.988 (0.962 – 1.014)	0.356
Comorbid psychosis	1.071 (1.045 – 1.098)	<0.001

## Discussion

An added burden to epilepsy is psychiatric comorbidity [12-13]. Our study shows that the prevalence of psychiatric comorbidity in patients with epilepsy is estimated to be 39.9%, which supports the previous studies that state that the prevalence rates of comorbid psychiatric conditions are 37% [14] and 29% [15] in epilepsy. Our study also found that psychiatric comorbidities are the most prevalent comorbidities as compared to other disorders in epilepsy patients and, thus, clinicians need to focus more on psychiatric disorders to improve hospital outcomes.

The most common comorbid psychiatric disorder that is associated with epilepsy is depression [16]. As per our study, depression is the most prevalent psychiatric comorbidity too, as it constitutes 13% of the comorbidities related to epilepsy. A recent study reported a significant dose-related linear relationship between depressive symptoms and Health-Related Quality of Life (HRQOL), such that the more frequent and severe the symptoms of depression, the poorer the patient’s HRQOL [17]. Depression seems to be affecting the quality of life of patients with epilepsy more than seizures, and they have a poorer treatment outcome [18]. As per our study, there are an estimated 51,885 total hospitalizations for epilepsy patients with comorbid depression, which is associated with the highest risk of a longer length of stay and greater inpatient total charges, which indirectly affects HRQOL. Comorbid depression for epilepsy patients should serve as a red flag for increased healthcare utilization and interventions to reduce the risk of poor hospitalization outcomes for comorbidity.

Alcohol consumption, one of five most important risk factors for the global burden of disease and disability [19-20], has been shown to be associated with epilepsy. Very little systematic information is available on comorbid alcohol and drug abuse in patients with epilepsy till now. Small quantities of alcohol do not pose a grave risk for increased epilepsy frequency. However, alcohol intoxication or withdrawal is usually associated with an increased risk of developing epilepsy [21]. Alcohol abuse and withdrawal seizures are related to generalized tonic-clonic seizures (GTCS), and alcoholics may progress into status epilepticus (SE) from their withdrawal seizures. As per our study, epilepsy with comorbid alcohol abuse had the highest risk of inpatient mortality as compared to other psychiatric comorbidities. The prevalence of alcohol abuse is low as compared to other psychiatric comorbidities; due to this, comorbid alcohol abuse is less focused, but seeing the results of our study, it confirms that though this comorbidity is less prevalent, its impact on inpatient mortality is significantly higher. Thus, comorbid alcohol abuse needs adequate management to improve the hospital outcomes of epilepsy patients.

Previous systematic review studies found that up to six percent of individuals with epilepsy have a comorbid psychotic illness and that patients have an almost eight-fold increased risk of psychosis [22]. As per our study, psychosis was the second most common psychiatric comorbidity (10.4%), after depression in epilepsy patients. Comorbid psychosis did not increase the risk of in-hospital mortality in comparison to other psychiatric comorbidities, but it was associated with increased risk of a longer length of inpatient stay and higher inpatient total charges in epilepsy patients. Similar to depression, this indicates that psychosis has adverse effects on HRQOL.

Psychiatric comorbid disorders are relatively frequent in epilepsy and often precede the onset of the seizure disorder. Their early identification and management are essential to avert the negative impact they exert on the patient’s quality of life as well as other serious morbidities and mortality risks. Unfortunately, psychiatric disorders in epilepsy remain underdiagnosed and, when identified, they are not treated optimally. Comorbid alcohol abuse needs to be focused on, as it is the only disorder among other psychiatric comorbidities that increase the risk of in-hospital mortality in epilepsy patients, as per our study. Comorbid depression and psychosis do not increase the risk of in-hospital mortality, but they worsen HRQOL in epilepsy patients by increasing the risk of the longer length of hospital stay and higher inpatient hospitalization charges.

 We used the NIS as a nationally representative sample of patients diagnosed with epilepsy. We applied sampling weights to generalize estimates for comorbidity prevalence. The inpatient outcomes are generalizable to a bigger population than the sample studied. Using the NIS data set, there was a large sample size because we included 397,440 epilepsy patients. This dataset is subject to minimal reporting bias, and all information is coded independently of the individual practitioner, making it a potentially more reliable source. This is the first study, to our knowledge, that reports the impact of various psychiatric comorbidities in epilepsy patients regarding hospital outcomes and in-hospital mortality. The limitation for using hospitalization (and not patient) as the unit of analysis is that it does not translate to generalizability for all patients with epilepsy. There may have been underreporting of chronic comorbidities in the NIS data because the administrative database was used. Hence, clinical data were not incorporated in the data source. We recommend that future research examine the influence of psychiatric comorbidities with clinical data.

## Conclusions

Among a large sample of hospitalizations for epilepsy, we observed four distinct psychiatric comorbidities that were associated with substantial and significant differences in the risk of inpatient death, length of stay, and inpatient cost. We assert that these findings indicate that psychiatric disorders are influential factors that must be considered in models of HRQOL in epilepsy. Further, efforts to improve HRQOL and lessen the burden of epilepsy requires a greater emphasis on the early diagnosis and treatment of comorbid psychopathology. To date, these efforts have focused exclusively on depression. Other psychiatric comorbidities like alcohol abuse and psychosis in epilepsy can exert significant adverse effects on HRQOL and hospitalization outcomes and should be included in recognition and treatment efforts. The focus of future research and the target of future interventions should aim at reducing the risk of inpatient death and health care utilization.
